# 240 Does fall prevention also have to hinder physical activity?

**DOI:** 10.1093/eurpub/ckae114.162

**Published:** 2024-09-26

**Authors:** Eva Ekvall Hansson, Agneta Malmgren Fänge, Steven Schmidt

**Affiliations:** Lund University, Medical Faculty, Dep of Health Sciences, Lund, Sweden; Lund University, Medical Faculty, Dep of Health Sciences, Lund, Sweden; Lund University, Medical Faculty, Dep of Health Sciences, Lund, Sweden

## Abstract

**Purpose:**

Physical activity is effective for preventing falls and improves physical and mental health, increase function and quality of life. Regarding people at high risk of falling, such as residents at nursing homes, global guidelines recommend individually adapted exercise programs to prevent falls. These guidelines are not followed. We aimed to map out physical activity among people living at a nursing home. To our knowledge, this is the first study measuring physical activity 24 hours per day among residents at a nursing home.

**Method:**

In this pilot study, persons who lived at one nursing home and could walk without a walking aid wore an Inertial Measurement Unit for 24 hours per day for 3 months. Main outcome measure was steps per day. For each participant, the mean steps per day during the period was calculated and for the total group, the median value of the mean steps per day was calculated.

**Results:**

The participants (n = 5), three women and 2 men, 72 to 98 years old, walked in a median of 65 steps per day (10-356).

**Conclusion:**

In Sweden, instead of following guidelines, technical solutions that monitor if a person tries to move are introduced at nursing homes, often in the form of web cameras. This means that falls are prevented by hindering physical activity and making sure the residents do not move at all. Our study shows that people living in nursing homes can have a very low level of physical activity. We call on all responsible organizations to initiate activities and efforts that enable rather than hinder physical activity. For example, use technology that promotes physical activity, build gyms located in nursing homes, use Virtual Reality to make the bike ride seem to take place outside in a beautiful landscape, and employ physiotherapists who can do balance-training exercises on an everyday basis.

